# AG1®, a foundational nutrition supplement, closes nutrient gaps in healthy resistance-trained adults

**DOI:** 10.1080/15502783.2025.2550174

**Published:** 2025-09-02

**Authors:** Adam M. Gonzalez, Philip A. Sapp, Jeremy R. Townsend, Caitlyn Edwards, Trevor O. Kirby, Lara Nyman, Ralph Esposito

**Affiliations:** a Hofstra University, Department of Allied Health and Kinesiology, Hempstead, NY, USA; b Research, Nutrition, and Innovation, AG1, Carson City, NV, USA; c Concordia University Chicago, Health & Human Performance, River Forest, IL, USA; d New York University-Steinhardt, Department of Nutrition, Food Studies and Public Health, New York, NY, USA

**Keywords:** Supplement, diet quality, micronutrients

## Abstract

**Background:**

Inadequate intake of micronutrients is common even among active, resistance-trained individuals. Supplementation with a foundational nutrition supplement (AG1®) may promote adequate intake of key micronutrients, close nutrient gaps, and improve diet quality.

**Methods:**

In a randomized, double-blind, placebo-controlled crossover design, this study evaluated the effect of daily AG1 supplementation on nutritional adequacy in 20 resistance-trained men (*n* = 10; 26.4 ± 6.5 y; 174.5 ± 9.8 cm; 85.8 ± 9.6 kg; 10.3 ± 7.9 y resistance training experience [RTE]) and women (*n* = 10; 26.9 ± 5.3 y; 163.8 ± 8.7 cm; 66.1 ± 13.0 kg; 9.1 ± 5.4 y RTE). Participants supplemented daily with AG1 or placebo for 14 days. Following a 2-week washout, participants crossed over to the other condition. Participants completed a 24-hour dietary intake assessment at the start (baseline) and end of each 14-day supplementation period (endpoint) using the Automated Self-Administered 24-Hour Dietary Assessment Tool (ASA24®). Nutrient gaps were defined as the difference between an individual’s reported dietary intake and established dietary targets [the Estimated Average Requirements (EARs) for specific nutrients]. Sixteen micronutrients were included in the assessment based on the following criteria: micronutrients were present in the study product, could be measured by ASA24®, and had an EAR. The combined nutrient gap score was determined by adding the nutrient intakes that met the EAR, with each micronutrient meeting the EAR counting as 1, for a total maximum score of 16. Nutrient values from AG1 or placebo were added to the respective endpoint diets, and a nutrient gap score was computed to determine the impact of each treatment. Data were analyzed using 2-way analysis of variance and Fishers’s LSD test for multiple comparisons.

**Results:**

All 20 participants completed baseline and endpoint recalls and were included in the nutrient gap analysis. At baseline, the AG1 and placebo groups met 13.9 ± 2.5 and 12.8 ± 2.8 out of 16 nutrient EARs, respectively with no significant difference between groups (*p* > 0.05). Following the intervention, AG1 significantly increased the total number of EARs met compared to placebo [2.8 (95% CI: 1.1, 4.4); *p* = 0.0011]. Within the AG1 group, 1.4 (95% CI: 0.3, 2.5) nutrient gaps were closed on average compared to −0.2 (95% CI: −1.3, 0.9) in the placebo group. Vitamins A, C, and E were the most common nutrient gaps filled by AG1 supplementation.

**Conclusions:**

AG1® consumption significantly improved nutrient adequacy by reducing nutrient gaps in healthy resistance-trained adults.

